# Framework to Define Structure and Boundaries of Complex Health Intervention Systems: The ALERT Project

**DOI:** 10.3389/fpubh.2017.00182

**Published:** 2017-07-28

**Authors:** Elena Boriani, Roberto Esposito, Chiara Frazzoli, Peter Fantke, Tine Hald, Simon R. Rüegg

**Affiliations:** ^1^Global Decision Support Initiative (GDSI), Technical University of Denmark, Kongens Lyngby, Denmark; ^2^National Food Institute, Technical University of Denmark, Kongens Lyngby, Denmark; ^3^External Relations Office, Istituto Superiore di Sanità, Rome, Italy; ^4^Department of Cardiovascular, Dysmetabolic and Aging-Associated Diseases, Istituto Superiore di Sanità, Rome, Italy; ^5^Quantitative Sustainability Assessment Division, Department of Management Engineering, Technical University of Denmark, Kongens Lyngby, Denmark; ^6^Vetsuisse Faculty, Section of Epidemiology, University of Zurich, Zurich, Switzerland

**Keywords:** food safety, food security, primary production, food chain, dairy chain, interdisciplinary, transdisciplinary, One Health

## Abstract

Health intervention systems are complex and subject to multiple variables in different phases of implementation. This constitutes a concrete challenge for the application of translational science in real life. Complex systems as health-oriented interventions call for interdisciplinary approaches with carefully defined system boundaries. Exploring individual components of such systems from different viewpoints gives a wide overview and helps to understand the elements and the relationships that drive actions and consequences within the system. In this study, we present an application and assessment of a framework with focus on systems and system boundaries of interdisciplinary projects. As an example on how to apply our framework, we analyzed ALERT [an integrated sensors and biosensors’ system (BEST) aimed at monitoring the quality, health, and traceability of the chain of the bovine milk], a multidisciplinary and interdisciplinary project based on the application of measurable biomarkers at strategic points of the milk chain for improved food security (including safety), human, and ecosystem health (1). In fact, the European food safety framework calls for science-based support to the primary producers’ mandate for legal, scientific, and ethical responsibility in food supply. Because of its multidisciplinary and interdisciplinary approach involving human, animal, and ecosystem health, ALERT can be considered as a One Health project. Within the ALERT context, we identified the need to take into account the main actors, interactions, and relationships of stakeholders to depict a simplified skeleton of the system. The framework can provide elements to highlight how and where to improve the project development when project evaluations are required.

## Introduction

Recent financial, economic, social, environmental, and health crises have led to the renewed recognition that collaborative approaches between disciplines are urgently needed to tackle such global challenges (2, 3). Consequently, the approach to emerging pandemics, as well as climate change, drug resistance, food and water security, and safety, has shifted from an interdisciplinary approach of experts, whereby experts collaborate across disciplinary boundaries, to a transdisciplinary approach that integrates society and science by including all potentially affected or otherwise relevant stakeholders (4–6). This transcends traditional boundaries and integrates knowledge and perspectives from scientific and non-scientific sources (2, 7). Many health communities have proposed transdisciplinary and systemic approaches with different focus points, such as EcoHealth, Global Health, Planetary Health, or Health in scaled Social–Ecological Systems (5, 6). The end goal is to have an additional instrument to improve the effectiveness of health intervention/care projects, thereby ensuring safety for humans, animals, and the environment alike (8).

The One Health approach, and ALERT as example, employed in a health intervention project that will be used in our manuscript, aims at simultaneously considering human and ecosystem health (9). Integration of multiple disciplines, sectors, stakeholders, living and inanimate elements yields highly complex constructs with varying dynamics at different scales. Although there is considerable literature describing the integrated approaches to health (10, 11), to the best of our knowledge, there are no recognized guidelines on how to evaluate to what extent the underlying integration as a principle contributes to address especially complex health problems, such as antibiotic resistance, outbreaks of highly infectious or non-communicable diseases, or ecotoxicology (12). There is a clear need for methods to represent and analyze such initiatives for management and evaluation purposes using a systemic and integrated approach (13).

The aim of the Network for Evaluation of One Health,^1^ a Transdomain action of the European Cooperation for Science and Technology, is to enable appropriate evaluations of One Health activities and hence comparison of initiatives as well as informed decision-making and resource allocation. Its conceptual framework includes the definition of the (One Health) system in which the initiative is implemented and the definition of the scale and boundaries of the system under evaluation. Human relationships, cultural behaviors, languages expressions, governance organizations, and constructive collaboration within interdisciplinary groups are all elements to be potentially included in a system. A possible way to visualize interactions and connections in and among different systems can be the system network approach.

We propose to employ a complex systems’ perspective to overcome the shortcomings of the traditional reductionist approaches (14–16). We consider “system thinking” as the process described by Whitehead et al. (17). These authors describe the “baseline lexicon of systems thinking” as being: descriptive scenarios, system boundaries, system stakeholders, scope of the analysis, type of system (state of system and life cycle of system), metrics, axiological components, observer effects, normative scenarios, indices of performances, and development alternatives (out-scoping, evaluating and ranking alternatives, interactions, iterating analysis, and leverage points). Occasionally, more appropriate elements can be added or other lexical components can be used.

In health, systemic techniques have been applied to work on problems such as obesity and epidemic diseases. WHO recommends some main techniques to identify points of interventions (leverage points) in complex health-related systems (3). The guideline differentiates building blocks of a health system such as health services, health workforce, medical technologies, financing, governance, and the main system goals like improved health, responsiveness, and improved efficiency.

The framework advocates a systemic overview of those building blocks to visualize synergies and the dynamic architecture. The main goal of the approach is to consider the effect of an intervention across as many major subsystems of the health system as possible. This process is initiated with a “stakeholders analysis,” where the interconnections and perspectives of each stakeholder are inventoried. This is overlaid with an open and transparent network of interventions and their possible consequences.

In a more generic manner, systems’ thinking has been applied in project evaluation to inform policy makers and executives for best resource allocation (18). The systemic approach is intended to design programs and policies that are aware of and prepared for possible unintended consequences and that integrate multiple stakeholder perspectives. The resulting framework or model should describe and predict the various ways in which a system might react to change.

For the evaluation of One Health interventions as an example, we have adapted the system thinking techniques to visualize the main elements, stakeholders, constrains, and internal dynamics. Furthermore, defining the system boundaries allows exploring the needs and gaps of a system. Once these elements are established, the system can be studied in a prospective and retrospective way, to optimize it or maximize benefits from interventions on it.

In this study, we propose a framework to describe and delimit One Health initiatives using ALERT as an example project as a first step toward evaluating them as complex adaptive systems.

Our framework will be a further instrument to be used alone or combined and used in synergies with other already existing frameworks. The right choice of a specific framework or a combination of different frameworks for analyzing different types of complex systems will have to be addressed in a separate effort and it will require specific expertise (in particular, socioeconomic background) that will collaborate with us in the future work.

## Materials and Methods

### Preliminary Considerations about Systems and Their Boundaries

Whitehead et al. (17) and Gibson et al. (19) define a system as “a set of elements so interconnected as to aid in driving toward a defined goal.” A system might include subsystems or a collection of systems. In other words, abstractions about systems and their constituent components can go to very high and very low levels of detail [intending level as a position in a scale or rank (20)] depending on one’s perspective and the purpose of the abstractions. The lack of specificity in defining what is a system vs. a subsystem or system component or element is one reason why all relevant stakeholders should be involved in defining the structure of a system. In public health, “systems are dynamic architectures of interactions and synergies,” where the elements of the system are also coming from social science (3). When working with such complex systems, the meaning of multiple perspectives, interactions, and boundaries must be understood, because each element can be potentially essential for identifying successful interventions. While the perspectives are determined by the stakeholders, these are at the same time players in the system and may become agents of change (3, 21).

Any observation, intervention or evaluation, faces the dilemma between focus and comprehensiveness, and to become operational, the system of interest must have an operational space that defines its limitations (21). Nevertheless, the environment of a system is important and should be well described to generate awareness of the wider context and to avoid missing potential external interactions. Real systems are dynamic and even geophysical boundaries change over time (22). We consider the dynamic of the system adopting an iterative process. If there are modifications in the inner scenario, we consider the modified scenario as new inputs and iteratively integrated it to the new analysis, as described by Gibson et al. (19).

### Elements of the System Definition Framework

In the next section, we define the elements contributing directly or indirectly to the system and system boundaries, i.e., the network of connected interactions that temporally close it, represent limits, and contribute to the overall structure of the system. We have then combined these elements to create the framework for defining and analyzing the structure and boundaries of a system and applied this framework on a health intervention project in the Section “Results.”

#### The Aim of a System

The aim should provide an answer to the question “Why are we looking at this system? Which are the problems, questions to solve?” The aim should help to investigate the way a system is used to solve a problem.

In the framework, we differentiate among the declared aim by the system and the observed, enacted, as well as the perceived aims. Each stakeholder may have a different perception of the declared aim and again each of them can have a different way to interpret how the system is performing in relation to its aim (23).

#### The System Space and Time, and Scale of Analysis

##### Space

This element identifies how the system extends geospatially, what is the geophysical environment, how large it is, and which ethno-political entities are involved (region, state, and nation). It also defines the scale of analysis that is of primary interest, individuals, households, groups or populations, etc., and finally how the different stakeholders are influenced by the spatial conditions.

##### Time

This element defines at which primary time scale is the system being observed, such as seconds, days, weeks, months, years, etc., and how the stakeholders are influenced by this time scale.

##### Interactions with Space and Time

This element defines the involvement of iterations and pathways along space and time dimensions.

#### Stakeholders and Actors

Stakeholders are entities affecting or affected by the system; they can be entities of different size between individuals, families, institutions, government agencies, etc. (24). More specifically, we can define primary actors as stakeholders who *act* on the system and secondary actors that can still partially interact with and modify the system. Examples of stakeholders of a health intervention system are farmers at the beginning of the milk chain, veterinarians checking the animals, dairies, food industry, toxicologists, chemists, veterinarians, biologists, and agronomists part of a research institute or a control institution.

In the framework, the information how the actors and stakeholders influence or are influenced by the system is specifically required.

#### The Systems Restrictions/Conditions—Boundaries

Which are the restrictions, conditions, and boundaries associated with a system? For example, a limited production due to regulations (e.g., the old milk quotas system in Europe), a closed market for a food due to regulations (e.g., raw milk consumption), or a cultural behavior that will limit a certain system to a group of individuals or animals (e.g., bovine milk consumption in certain Asian communities), relations of control among stakeholders and actors and relevant legal requirements, or constraints imposed by daily food production and market (e.g., quality systems must comply with production time), financial capability of primary food producers (e.g., calling for public incentives) or by sustainability aspects [e.g., the impact of the global milk production on the environment at the global level, i.e., in relation to the planetary boundaries (22)]. Such elements need to be potentially considered when defining the boundaries of a system in line with the system aim and goals. As defined by Senge (25), the description of system boundaries “considers what is improved, affected, or replaced by the system and, conversely, what affects the system under study, as the system changes and is changed by the environment.” Being able to well define the system space and its limitations helps in the definition of the system boundaries.

In the framework, constrains and boundaries are also related to the aim of the system. Understand how they interact with the system aims is important information to understand possible leverage points.

#### The Consequences (of Different Degree)

Consequences are the results of interactions in the system. They follow the path of interactions and stop at the system boundaries. Once the system and the system boundaries are defined, the consequences for that system are determined subsequently inside the system space. The “boundaries,” “externalities,” and “constraints” to the system should also be considered as surrounding and limiting the consequences to a certain degree (26). As an example, foods and food products are impacting directly human or animal health and indirectly (e.g., through the food chain or animal or human waste) ecosystem health.

In the framework, the consequences are related to the boundaries/constrains of the system, as described above.

#### The System Evolution

Following the work of Forrester (27): “there is not a single method, but an approach that uses a set of tools, to understand the behavior of complex systems over time designed to solve the problem of simultaneity (mutual causation).” Every real system is dynamically evolving, and this is why it is important to periodically reevaluate the system (interacting actors and stakeholders, restrictions, and consequences) and redefine it and its boundaries in an iterative way. The various processes of definition of the system and the problems that should be solved, the definition of a way to act/interact with a specific population/culture/disease/habit should be redefined periodically, because the system itself is constantly under change.

### The ALERT Project

The ALERT project^2^ has been used as an example on how to apply the proposed framework. ALERT is funded by the Italian Ministry for Economic Development and is based on the transfer of technical innovation and technological know-how, which emerged from public research in the field, to the actors in daily food production (primary food producers and food industry). ALERT is coordinated by the Italian National Institute of Health (ISS) and develops a new risk management framework based on recent technological advances to manage the bovine milk chain for improved product safety and quality. It exploits new biomarkers for (i) early detection of production anomalies, (ii) monitoring (and assessment) of effects of corrective actions undertaken as risk management measure, and (iii) for assessment of production improvements (e.g., feed changes).

ALERT involves the human health affected by the environment, animal health and ecosystem health, sectors, as well as the producing farmer community and their web of interactions, and other actors of the food chain up to the food industry and the consumers. It is transdisciplinary and multisectoral to provide space for innovation and harvest the benefits of such integrated approaches and can thus be seen as a One Health project. It further focuses the responsibility of primary producers in the European food safety framework (28). Milk is a food particularly interesting for One Health: it is an animal product, highly susceptible to toxic contaminants (29, 30), highly consumed by vulnerable consumers, and suited as sentinel matrix for environmental monitoring purposes (30). The description of the project system may also be of interest to primary productions in economically developing countries, where environmental conditions and restricted resources amplify both risks of contaminations and challenges for their prevention (29).

In Table 1, the ALERT response to the primary producers’ mandate for legal, scientific, and ethical responsibility in the EU food safety framework is described.

**Table 1:** ALERT project response to develop and translate invention (BEST) into practical innovation for One Health-related needs.

Primary producers’ mandate for legal, scientific, and ethical responsibility in the European food safety frame	ALERT: from invention (BEST) to innovation
**One Health-related needs**	**ALERT response**

**Figure 1:**
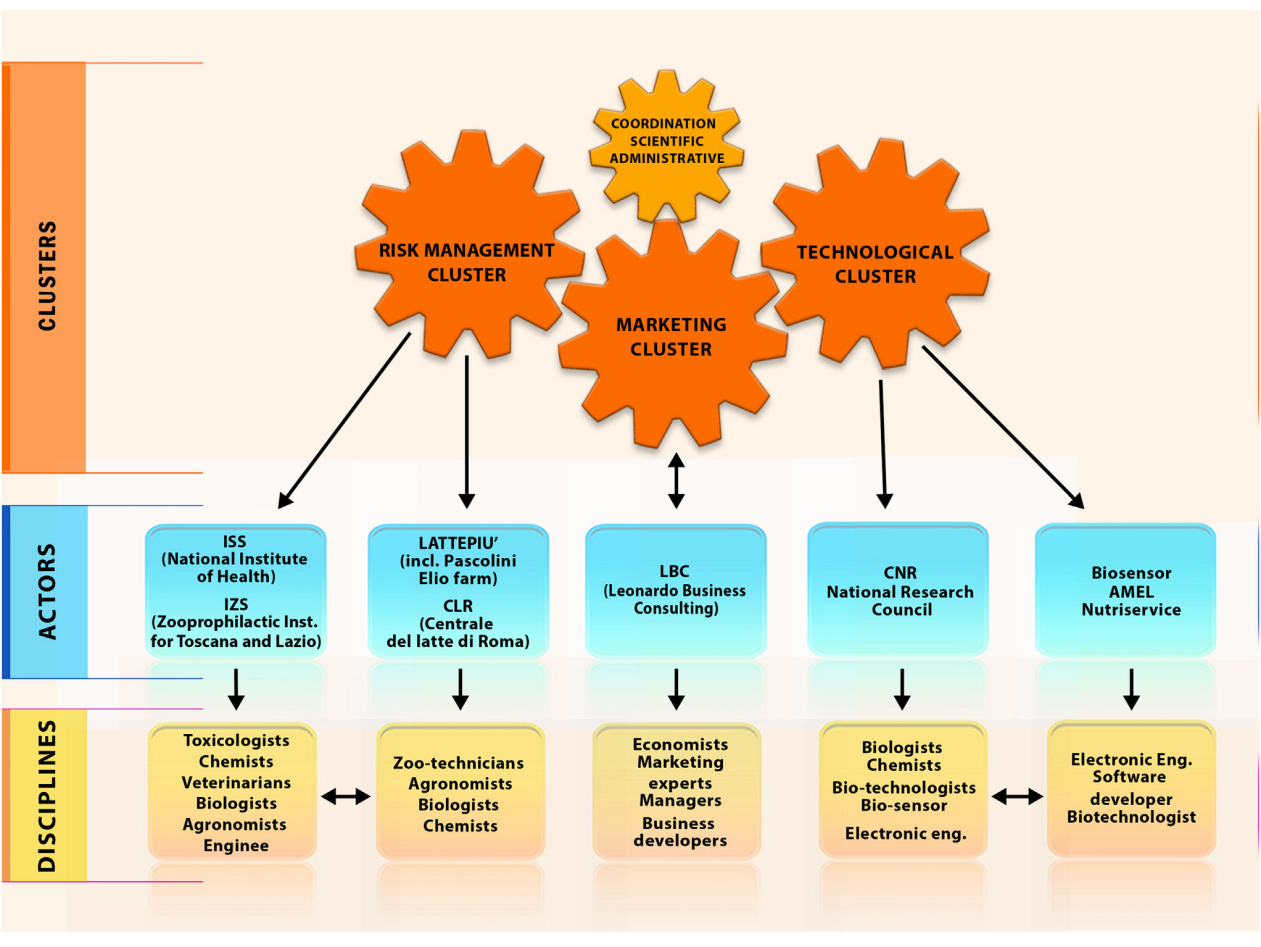
Institutional framework governing the ALERT project.

**Figure 2:**
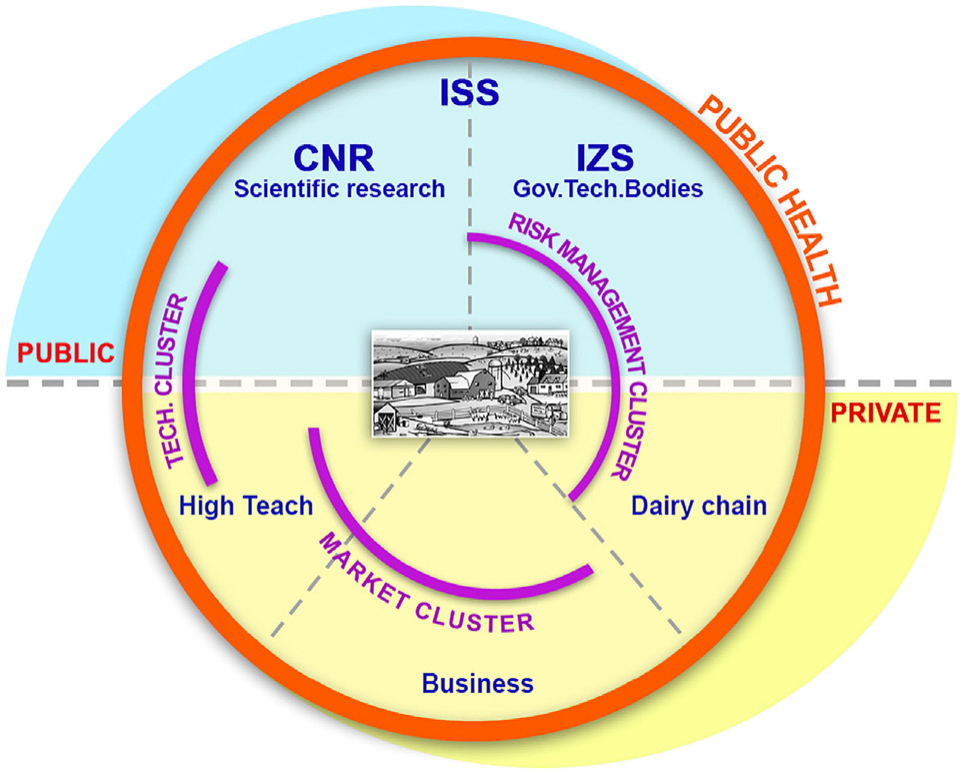
Interdisciplinary team in ALERT.

**Filling knowledge gaps**Besides the field of physiological, behavioral, and production and reproduction indicators for improving rearing management, strategies and performance of farmed animals (food security), food safety aspects need increasing attention by all food chain stakeholders. New zoonotic threats from foods of animal origin (i.e., from animals as food-producing living organisms) are a scientific topic with many knowledge gaps. Moreover, significant health-relevant know-how in different fields is scarcely integrated due to the different sectoral silos	The Hazard Analysis and Critical Control Points system is the on-enterprise strategy to control and manage the safety of food production process. ALERT develops new knowledge including biomarkers of unmanaged indicators of undesirable substances (chemical and microbial pollutants) and milk quality (milk composition, subclinical mastitis, and metabolomic markers) and updated risk analysis (risk assessment and management) in the supply chain, in different scenarios (e.g., economically developed and developing areas, clean and contaminated sites) through its *multidisciplinary team* (Figure 1) integrating *different silos* (Figure 2)

**Optimization of resources**Besides periodic (e.g., annual) controls, to date, self-monitoring plans of dairy enterprises consider only limited systematic activities. Significant resources invested in official control require increased cost-effectiveness through science-based criteria	ALERT designs new strategy to implement the enterprise early risk management system including toxicological risks and based on early warning. ALERT develops control charting of (grids of) *early biomarkers* based on (and feeding) *risk analysis* in food production

**Acceptability**Based on the end users’ perspective, the proactive role of primary food producers in building food safety benefits of tools already in use at farm level (such as control charting) and of standard values taken from the history of the enterprise	ALERT proposes: (i) a *two-lane (top-down and bottom-up) system* for food safety: field biomarkers in sentinel living animals and sentinel food matrices/animal excretion (milk) are suited to integrate the consolidated European system for Official control (fixing maximum residue levels, unacceptable contaminants, and tolerated contaminants at certain maximum levels) with field monitoring of farms environments to reduce vulnerability to unexpected events (ii) to complement the sophisticated and expensive laboratory instruments and techniques of official control with cost-effective probes working daily during farm operations to monitor *invariability* of significant farm quality and wholesomeness parameters as well as deliberate changes/improvements of production components (e.g., effects of feed on milk nutritional quality) (iii) historical trend in quality and safety parameters is relied upon as internal standard. Indeed, instead of burdening producers with closer external control activities and standards, BEST *monitors anomalous variations in historical enterprise’s trend* rather than official thresholds (iv) an early risk management system (based on cost-effective technology and self-monitoring plan) eventually allowing timely corrective action and avoiding both *food losses and food waste*

**Science in the farm scenario**Farm environmental conditions, daily need of food production, as well as farmers limited capacities and resources require highly innovative technology and risk analyses know-how. Animal physiology as well as the complexity of a “living” matrix like milk increases the scientific challenges of the monitoring purpose. On the other side, farmers’ expectations from Precision Livestock Farming already proved how farmers welcome the use of technology	ALERT proposes: (i) on-farm *robust technology* (without transferring samples to external laboratory) (ii) a *self-instructed system* (iii) a “*data in/acoustic-luminous signal out*” technology is easily handled and interpreted by unskilled operators (iv) *holistic/metabolomic* approach to monitor animal excretion fluid (milk) of individual animals

**Fair value chain**Agro-zootechnic enterprises are the most critical and strategic sites for the prevention of environmental adverse effects on health. Indeed, most of the environment-food web of interactions (both beneficial and noxious) occurs here (e.g., relevant to environmental quality, animal health/welfare, and farm management). To date (i) resources invested in official control prevention plans (including traceability) are scarcely focused on early warning in agro-zootechny; (ii) farmers are the most suffering group of food business operators	ALERT proposes: (i) farmers *empowerment*: indeed, farmers are a key building block of public health (ii) monitoring farm’s vulnerability to unexpected events (iii) a stable technological platform (BEST) to *interface* dairy enterprises *with scientific research* (iv) tools to increase citizens’ trust in milk primary production (v) social innovation [Start Cup prize for social innovation MILKNET (31)]

**From farm to fork**The *from farm to fork* approach implemented by the EU strategy builds a chain of responsibility (and value thereof) along the different segments of the food chain. It also promotes a common approach by the food chain actors, including innovative and sharable technologies and risk management strategies	ALERT designs: (i) a new strategy for risk management along the entire food chain, i.e., a *centralized system of BEST devices* along the whole chain, including environment/farm interface (watering, milking, and raw milk harvesting) and milk factory (milk exiting tank-lorry, exiting pasteurization, exiting microfiltration, and at packaging) (ii) food chain traceability along the different production segments The food safety system benefits from food operators empowered in their knowledge of the food production chain

#### ALERT Institutional Framework

ALERT requires the establishment of an institutional setting matching different silos like public vs. private bodies; public health vs. fundamental research; food industry vs. high-tech industry; risk analysis vs. marketable technologies; scientists vs. food producers; and scientists vs. citizens/consumers.

ALERT relies on the integration between three clusters or pool of actors:

(a) A “Risk Management Cluster” including:
  - Istituto Superiore di Sanità (ISS), i.e., the Italian National Institute of Health, is the leading technical-scientific body of the Italian National Health Service, with top-level expertise in risk analysis (from risk assessment to formulation of scientific options for risk management) of food chains (Department of Food Safety, Nutrition and Veterinary Public Health), public health (Department of Environment and Health), prevention of non-communicable diseases and relevant technologies development (Department of Cardiovascular, Dysmetabolic and Aging-Associated Diseases). *Disciplines/expertise* deployed: anthropologists, biologists, chemists, engineers, statisticians, toxicologists, and veterinarians.
  - Istituto Zooprofilattico Sperimentale of regions Lazio and Toscana, i.e., public institute with top-level expertise and governmental commitment in the protection of food chain wholesomeness and animal health and welfare in the network of IZS regional institutes. *Disciplines/expertise* deployed: agronomists, biologists, chemists, and zootechnicians.
  - Lattepiù, i.e., leading enterprises in milk production, including the Pascolini Elio dairy farm, and milk transport and storage system. *Disciplines/expertise* deployed: farmers and livestock staff.
  - Centrale del Latte di Roma (CLR), i.e., leading regional milk industry. *Disciplines/expertise* deployed: biologists and chemists.
  Lattepiù and CLR are also the final end users of the ALERT products.
(b) A “Technology Cluster” including:
  - Consiglio Nazionale delle Ricerche (CNR, Institute on nano-structured materials), i.e., public institute with top-level expertise/commitment in technological innovation, research and technology transfer. *Disciplines/expertise* deployed: biologists, chemists, and biotechnologists.
  - Amel, Biosensor, Nutriservice, i.e., enterprises with complementary expertise in the setting, optimization, miniaturization, and robotization of (bio) probes systems as well as the development of management software and electronic systems. *Disciplines/expertise* deployed: biotechnologists, electronic engineers, and software developers.
(c) A “Marketing Cluster” involving different expertise in marketing strategies, strategic partnering at industrial level, as well as dissemination of pre-industrial research deliverables of the Leonardo Business Consulting. *Disciplines/expertise* deployed: economists, marketing managers, and business developers.

Indeed, the primary producers’ role in the EU food safety frame calls for transdisciplinary work as it is shown in Figure 2.

## Results

The proposed framework (Table 2) provides an overview on elements and relationships of the system in which ALERT operates (Table 3). By employing a structured process to define system elements and boundaries, the system representation is developed from the various stakeholders’ perspectives and sets the system boundaries. In analyzing the three aspects of contextualization, relationships and evolution, one gains an understanding of static and dynamic properties of the system.

**Table 2:** Framework for identifying a system and its boundaries.

	**Element**	**Main question**	**Secondary question**	**Tertiary question**
	**Definition of**	**Contextualization**	**Actions/relationships**	**Evolution/dynamics**

1	Aim	Why I am looking at this system? Which are the questions/problems I want to solve by using the system?	What is the declared aim of the system and what is the enacted aim of the system. Is the aim perceived differently by stakeholders?	What are the declared and enacted aims at the onset of the evaluation and do they change as the system evolves?

2	Actors/stakeholders	Which are the main actors/stakeholders? How are they affected by the system and/or how do they affect the systems?	How do actors influence/modify the system to achieve the aim?	Do the actors change their activity and behaviors because of the system evolution (new trade-offs)?

3	System space and time	Which geographical and political space does the system occupy (e.g., geography/area/countries involved)? Which is the most important time scale for observing the system (e.g., months and years), and what is the primary level of analysis for the evaluation of the system (e.g., individuals and family, population)	How are these dimensions connected with the declared aim of the system?	As the system evolves, how do these aspects change?

4	Restrictions/conditions/boundaries	What are the main restrictions/conditions/boundaries of the system? Are there constraints coming from the system’s external surroundings?	How do these restrictions/conditions/boundaries interact with the system aims?	Do these restrictions/conditions/boundaries change as the system evolves?

5	Consequences	What are the consequences of the system (outputs/results/products)?	Are these consequences bound by the system boundaries?	Are these consequences change as the system evolves?

**Table 3:** The system and system boundaries applied to the ALERT project as an example.

Step	Element	Main questions	Secondary questions	Tertiary questions
		
		Contextualization	Actions/relationship	Evolution/dynamics
**1**	**Aim**	**Why I am looking at this system? Which are the questions/problems I want to solve by using the system?**	**What is the declared aim of the system and what is the enacted aim of the system. Is the aim perceived differently by stakeholders?**	**What are the declared and enacted aims at the onset of the evaluation and do they change as the system evolves?**

		Based on European scientific and policy milestones (32), system was built to support primary producers in their mandate for legal, scientific, and ethical responsibility in the European food safety frame Stakeholders like food industry and bank systems recognize the need of field technologies and approaches for food safety in primary production: START CUP CNR-IlSole24Ore Prize for the best high-tech business idea for Social Innovation coming from public research (2011); MONTANA (meat industry and Cremonini group) Prize for Research in the Food sector (2011)	ALERT answers to identified One Health-related needs (Table 2) by combining different silos like public vs. private bodies; public health vs. basic research; food industry vs. high-tech industry; risk analysis vs. marketable technologies; scientists vs. food producers; and scientists vs. citizens/consumers (Figure 1). The aim and the stakeholders’ role are specified in Table 1 and Figures 1 and 2 ALERT points at defining and implementing toxicological risk and non-communicable diseases in One Health: so far the application of One Health has been limited to microbiological risk and infectious diseases	ALERT aims at establishing a frame for long-term bottom-up and top-down collaboration through both an open technological platform (i.e., able to improve its detection capability by hosting new probes made available by the scientific community) and an innovative two-lane system for food safety (Table 1) **SMEs, food chain, and institutional/research stakeholders have different vision of risks and benefits, based on different needs, mission and vision (as discussed in the text, Table 1; Figures 1 and 2) (a)**

**2**	**Actors**	**Which are the main actors/stakeholders? How are they affected by the system and/or how do they affect the systems?**	**How do actors influence/modify the system to achieve the aim?**	**Do the actors change their activity and behaviors because of the system evolution (new trade-offs)?**

		Actors (as specified in Figure 2) cover the range of public institute with top-level expertise/governmental commitment in food chain protection and technologies certification, public institute with top-level expertise/governmental commitment in the protection of food chain wholesomeness and animal welfare, public institute with top-level expertise/governmental commitment in technological innovation and transfer; leading regional enterprises in milk production, transport and storage; enterprises with complementary expertise in the setting, optimization, miniaturization and automation of (bio)probes systems as well as the development of management software and electronic systems; expertise in marketing strategies, strategic partnering at industrial level, as well as dissemination of pre-industrial research Relevant disciplines and the way they affect the system are detailed in Figure 2	Single enterprises of the milk chain **can adopt (b)** new self-monitoring strategies to minimize milk losses and waste as well as to optimize milk safety, nutritional value, and wholesomeness The chain of enterprises **can adopt** (b) new strategies to improve milk chain traceability Public Institutes that have the mission of securing a high level of safety of food products and food productions can update (b) tools and strategies based on modernized primary prevention plans **SMEs, food chain, and institutional/research stakeholders have different vision of risks and benefits, based on different needs, mission and vision. In particular:** – **attitude toward non-traditional approaches to protect food-producing animals and food productions: scientific research approach vs. market-driven food production needs;** – **availability to long-term investment: small-medium enterprises mainly depend on short-term economical benefits do to chronic constraints (d)**	**Awareness of toxicological risks and One Health in the food chain is increasing at both enterprise and scientific community levels (g)**

**3**	**System dimensions (space and time)**	**Which geographical and political space does the system occupy (e.g., geography/area/countries involved)?** **Which is the most important time scale for observing the system (e.g., months, years), and what is the primary level of analysis for the evaluation of the system (e.g., individuals, family, and population)**	**How are these dimensions connected with the declared aim of the system?**	**As the system evolves, how do these aspects change?**

		ALERT (2012–2017 project duration) focuses on a relatively large-sized dairy farm of Central Italy (and neighboring farms), and a main bovine milk chain in central Italy (Lazio region) Starting from cost/benefit assessment for all actors in the milk chain, ALERT evaluates the possible impact of the BEST on costs and marketability of milk products. ALERT assesses the value attributed by the consumers to a new brand/logo for the improved food chain control process	**ALERT outcomes can be applied (eventually with appropriate revision in certain milieu such as economically developing areas and contaminated sites) to other regions and nationwide (c)**	Increasing know-how in the chemical/toxicological (emerging) risk assessment Technological solutions, materials and methods change over time: an ALERT web platform collect census data of international probes that could be hosted by the BEST device Increasing consumers’ awareness of food safety long-term impact on health Increasing power of rearers’ Associations and consortia Through Expo 2015 (Milano, Italy), a unique event of knowledge of the food market and its needs in terms of technologies, ALERT gathers the needs of the national and international markets, and periodically update all relevant possible new stakeholders

**4**	**Restrictions/conditions/boundaries**	**What are the main restrictions/conditions/boundaries of the system? Are there constraints coming from the system external surroundings?**	**How do these restrictions/conditions/boundaries interact with the system aims?**	**Do these restrictions/conditions/boundaries change as the system evolves?**

		In the specific Italian scenario characterized by a high degree of one health in the institutional setting (veterinary health and food safety both under the Ministry of Health), main constraints are mainly relevant to: **Delayed ripeness worldwide and in different silos** (d) on toxicological risks in One Health (33, 34) **Need to strengthen sustainable food safety policies** (d) worldwide for primary prevention of transgenerational risks in the food chains (33, 35) from the technical viewpoint, ALERT proposes strengthening/modernizing the self-monitoring system to integrate/empower the two-lane system for food safety. This calls for investment (personnel, time, and materials) to set up a new organization flow during routine daily food production	**Limited confidence in the acquisition** (d) of new knowledge through non-traditional approaches vs. market-driven food production needs **Limited policies facilitating** (d) proactiveness toward emerging risks **Perceived different attitude toward the aim, in particular private vs. public institutions (d)**	Awareness on the importance of acquiring new knowledge through non-traditional approaches vs. market-driven food production needs Increasing movement to implement policies for facilitating proactiveness toward emerging (chemical/toxicological) risks in the farm

**5**	**Consequences**	**What are the consequences of the system (outputs/results/products)?**	**Are these consequences bound by the system boundaries?**	**Are these consequences changing as the system evolves?**

		Development of new field technology, decrease the vulnerability to unexpected events, increase the preparedness to emerging (chemical/toxicological) risks, reduce food losses and waste (risk of product recalls from the market and related costs of food destruction along with damage to the enterprises commercial image). **Turn upside down the responsibility to the primary food producer (and role in the value chain therefore) (e)**	Yes	**Dynamics mainly pivot on different interests in the system, from public health actors pointing at advancements in the protection of human health and the environment (including animals), food chain actors at the balance between needs of food security and food safety, and small-medium enterprises mainly depending on short- term benefits. (f)**

### System Identification Framework

A framework of the main definition steps to identify a system and its boundaries is summarized in Table 2 (below).

The framework is divided into three questions. Main questions are the ones that help to contextualize the system; secondary questions help to define the relationships among actions and finally the tertiary questions concern the evolution of the system.

These questions are made for each of the element described in the Section “Materials and Methods,” namely: (1) aim of the system, (2) system dimension (space and time), (3) actors–stakeholders, (4) restrictions/conditions/boundaries, and (5) consequences.

### Application of System and System Boundaries Framework to ALERT

The system and system boundaries framework is applied to the example ALERT to identify its gaps and weaknesses and relevant possible solutions (Table 3).

Using the framework, we can evidence some main arguments, highlighted in bold in Table 3 and summarized in Box 1. For clarity, we have associated a letter to each highlighted point in Table 3 and reported in Box 1.

Box 1 **Main results after the application of system and system boundaries framework to ALERT.**

The aim of the system is not interpreted in the same way by different stakeholders (a, d)
Stakeholders from different backgrounds and disciplines miss a harmonized collaboration to fulfill the aims of the project in the most productive way (b, d)
It is shown from the space and dimension elements that the system has potential to be applied at a larger scale (e.g., other regions and nationwide) (c)
Along the restrictions/constrictions/system boundaries, two main aspects (market-driven or daily tasks vs. importance of acquiring new knowledge through non-traditional approaches, and missing policies facilitating proactiveness at the farm) lead to the evolution scenario of weaknesses in synergizing activities between partners (d)
The consequences follow and detail the fact that the project aims to give more responsibility to the primary food producer and the different stakeholders approach this task with limited synergies (e)
The evolution of the system depicts needs and constrains in the future: namely, short-term/long-term effects along the awareness of toxicological risk/One Health approach in the food chain (g)

## Discussion and Conclusion

Using a structured framework that defines the system, its stakeholders, its boundaries, and its evolution helps in showing the situation as it is conceived, the stakeholder roles, the relationship, and recurrence of the system components. Furthermore, in line with a system thinking approach, we can observe the “leverage” points, described in the Section “Results,” that can modify the system evolution, for example, improving its performances, along a time line.

This type of project system and project system boundary analyses helps to have an overview on the project, without missing possible important connections within aim, expertise, business, and development factors (36).

To be able to improve the performance of a project, it is often required to not get “unwanted surprises” about its behavior, so to be able to follow almost predictable results, in line to the aim of the project. Considering that the system properties and behaviors are *per se* unpredictable along the project evolution, it is important to be able to understand why there may be a divergence from the wanted aims and the actual proceeding of the project.

Having such a project system description framework helping to connect the various elements of the project, also the little ramifications of a network of interactions among activities/performances/roles/results, gives the possibility to interpret the aim of the project in its real evolution and dynamic, as shown in Table 3. The strategy to defragment a project into such a framework may lead to the possible uses of the framework reassumed in Box 2.

Box 2 **Summary of possible use of the framework.**

To find possible representative information from the network of connections among aims, causes, consequences, and results of a project.
To prove the role of the different actors along the time line of the project, causes and consequences of their behaviors, and their points of view/background. This will help in getting a meta-perspective to evaluate a project. A further development of this basic framework can be to ask different stakeholders to apply the framework to the same project. Because their perspectives differ, such an analysis would further highlight synergies and antagonisms allowing for improvement.
To monitor progress along the project implementation phase (e.g., first and second year).
To compare and evaluate projects’ impacts and to measure their progress and compliance with the aim.

Different points are evidenced in the system and system boundaries analyses for ALERT example, in particular from Table 3 and Figure 2.

The aim of the project “to support primary producers” is maintained along the description of the system in the framework but many elements are competing with the main aim (see Table 3).

It is worth mentioning how the core aspect of the ALERT project, i.e., the emerging role of toxicological risk in the onset of diseases in the context of One Health (33, 34), is both the key and the “problematic” aspect of ALERT as showed in Box 1.

The increasing movement worldwide on the need of primary prevention measures to protect communities from non-communicable diseases as well as of sustainable food safety policies for primary prevention of transgenerational risks in the food chains (33, 35) is expected to modify the system in the medium term.

Indeed, the increasing consumers’ awareness of food safety long-term impact on health implies a growing demand of safer and safer products, along with the protection of the environment. In this context, policies facilitating primary food producers in their proactive roles are crucial (37).

The integrated analysis of the system and its boundaries including stakeholders, etc. highlights the importance of the collaboration within different disciplines, stakeholders, methodologies, expertise, etc. To allow the smooth project implementation taking into consideration the evidenced needed for interaction/collaboration/links among stakeholders and disciplines, governance mechanisms should be defined. However, integrating health aspects of humans, animals, and the environment is often not sufficient to identify relevant trade-offs, potential burden shifting, and undesired consequences in a system change, e.g., *via* an intervention. To address this issue, a more comprehensive approach is required where in addition to health- and risk management-related aspects also sustainability related aspects are considered along the entire system life cycle. Combining risk and sustainability aspects in a consistent manner to provide a more reliable decision support of health intervention and various other systems is proposed by the Global Decision Support Initiative (GDSI^3^).

In conclusion, a system view of a complex project and the definition of its boundaries help in understanding the way how to structure and optimize a system and actions (e.g., health interventions) within that system: how to integrate different expertise, meet different needs, mission and vision, and improve communications. This framework is a good way to have a concise, common overview of the most important elements that run a system now and in the future, considering the several externalities and impacts generated, but also to identify current system limitations.

## Author Contributions

All the authors contributed to the design of the work, to the acquisition and interpretation of data for the work, and to revise it critically and all the authors approved the final version to be published.

## Conflict of Interest Statement

The authors declare that the research was conducted in the absence of any commercial or financial relationships that could be construed as a potential conflict of interest.

## Footnotes

^1^http://neoh.onehealthglobal.net/.

^2^www.alert2015.it.

^3^http://gdsi.dtu.dk.
